# Draft Genome Sequence of Janthinobacterium sp. Strain ROICE36, a Putative Secondary Metabolite-Synthesizing Bacterium Isolated from Antarctic Snow

**DOI:** 10.1128/genomeA.01553-17

**Published:** 2018-04-12

**Authors:** Cecilia Chiriac, Andreea Baricz, Cristian Coman

**Affiliations:** a NIRDBS, Institute of Biological Research, Cluj-Napoca, Romania; b Molecular Biology and Biotechnology Department, Faculty of Biology and Geology, Babeş-Bolyai University, Cluj-Napoca, Romania

## Abstract

The draft genome assembly of Janthinobacterium sp. strain ROICE36 has 207 contigs, with a total genome size of 5,977,006 bp and a G+C content of 62%. Preliminary genome analysis identified 5,363 protein-coding genes and a total of 7 secondary metabolic gene clusters (encoding bacteriocins, nonribosomal peptide-synthetase [NRPS], terpene, hserlactone, and other ketide synthases).

## GENOME ANNOUNCEMENT

Rod-shaped, motile, Gram-negative bacteria belonging to the genus Janthinobacterium have been isolated from various environments in temperate and cold regions of the world, including cold Alaskan and Antarctic soils (1, 2), glacial lakes (3), alpine glacier cryoconite (4), Antarctic supraglacial streams (5), pristine groundwater (6), and the skin of amphibians (7). The species of this genus synthesize secondary metabolites with exceptional antibacterial, antifungal, antiviral, and antiprotozoal activities (3, 8, 9) and are considered a very promising source of novel pharmaceutical compounds.

Janthinobacterium sp. strain ROICE36 was isolated from snow samples collected from King George Island in West Antarctica during the Romanian Governmental Scientific Expedition in Antarctica—ROICE 2015. Microorganisms from extreme environments are a prolific source of diverse bioactive metabolites, which provide the majority of the most important products, like bacteriocins and antibiotics, in use today in the pharmaceutical industry. Pure cultures of the strain were obtained after plating of serial dilutions on solid Reasoner’s 2A (R2A) medium. The samples were incubated aerobically at 10°C under continuous illumination. Single colonies were selected based on morphology and/or pigmentation and restreaked several times until pure cultures were obtained. Janthinobacterium sp. ROICE36 showed the highest 16S rRNA gene sequence similarity with Janthinobacterium lividum strain DSM 1522^T^.

Genomic DNA extraction from Janthinobacterium sp. ROICE36 was carried out using the ZR soil microbe DNA kit (Zymo Research, USA), according to the manufacturer's instructions. Whole-genome sequencing was performed at Microsynth (Balgach, Switzerland). The sequence library was prepared with the Nextera XT DNA library preparation kit, with the sequencing step being performed on an Illumina MiSeq platform (∼250-bp paired-end reads). The adapter sequences and low-quality regions were filtered out with Trimmomatic software (10), and the final data set contained 2,294,412 high-quality paired-end reads. *De novo* assembly was performed on the error-corrected reads with AbySS (11), Velvet (12), and SPAdes (13) bioinformatics tools; the SPAdes software produced the best assemblies, which were further evaluated with QUAST (14). The final assembly contained 207 contigs, with a total genome size of 5,977,006 bp and a G+C content of 62%. The largest contig had 238 kbp, and the *N*_50_ value was 65,568 bp. The genome was annotated using the Rapid Annotations using Subsystems Technology (RAST) server (15), which showed 5,363 protein-coding genes (CDSs), 81 tRNAs, and 1 transfer-messenger RNA (tmRNA). The RNAmmer bioinformatic tool was used to determine the number and sequences of the rRNA genes, resulting in one large subunit (LSU) rRNA, one small subunit (SSU) rRNA gene, and eight 5S rRNA genes. From the total number of CDSs, 2,470 genes were included in RAST subsystems, of which, 16 were involved in osmotic stress response and osmoregulation and 2 in cold stress adaptation (CspA protein family); 71 genes have roles in protection against oxidative stress. Additionally, 107 genes providing resistance to antibiotics (e.g., beta-lactams and fluoroquinolones) and toxic compounds (e.g., Cd, Co, Zn, As, and Cr compounds) were identified.

*In silico* prediction of secondary metabolic gene clusters was performed using the Antibiotics and Secondary Metabolite Analysis Shell 3.0 (antiSMASH 3.0) Web-based pipeline (16), revealing seven gene clusters in the genome of ROICE36 (encoding bacteriocins, nonribosomal peptide-synthetase [NRPS], terpene, hserlactone, and other ketide synthases).

### Accession number(s).

The whole-genome sequence of Janthinobacterium sp. strain ROICE36 has been deposited at DDBJ/EMBL/GenBank under the accession number PEBS00000000.
